# Predictors of health management participation in community-dwelling older adults: a cross-sectional study based on the health belief model

**DOI:** 10.3389/fpubh.2026.1797890

**Published:** 2026-06-11

**Authors:** Rong Huang, Guangyi Luo, Tao Jiang

**Affiliations:** 1 School of Humanities, Zhuhai City Polytechnic, Zhuhai, China; 2 School of Humanities and Management, Guilin Medical University, Guilin, China

**Keywords:** community health, cross-sectional study, Health Belief Model (HBM), health management behavior, older adults, self-efficacy

## Abstract

**Objective:**

This study aimed to assess the current status of health management cognition, health beliefs, and behaviors among community-dwelling older adults in Nanning, China, and to identify factors influencing their health management behaviors using the Health Belief Model (HBM).

**Methods:**

A descriptive cross-sectional study was conducted from March to June 2024. A convenience sample of 250 older adults (≥60 years) was recruited from an urban community in Nanning. Data were collected through face-to-face interviews using a structured questionnaire, including sociodemographic characteristics, health management cognition and behavior items, and a culturally adapted Health Belief Scale (10 items, five dimensions). Reliability (Cronbach's α = 0.81) and construct validity (*CFI* = 0.94, *RMSEA* = 0.05) of the scale were confirmed. Statistical analyses included descriptive statistics, *t*-tests, ANOVA, Spearman's correlation, and binary logistic regression (SPSS 26.0).

**Results:**

The overall mean health belief score was 3.42 ± 0.68. Perceived benefits (3.85 ± 0.72) and self-efficacy (3.12 ± 0.81) were the highest and lowest-scoring dimensions, respectively. Only 54.4% of participants had ever received health management services. Binary logistic regression revealed that higher educational level (OR = 1.38, 95% CI*:* 1.01–1.90), higher monthly income (OR = 1.32, 95% CI*:* 1.01–1.72), presence of chronic disease (OR = 1.52, 95% CI: 1.03–2.23), stronger perceived benefits (OR = 1.62, 95% CI: 1.09–2.40), and greater self-efficacy (OR = 1.68, 95% CI: 1.10–2.56) were independently associated with higher likelihood of engaging in health management. Perceived susceptibility, severity, and barriers were not significant predictors in the multivariate model.

**Conclusion:**

Health management behaviors among older adults in Nanning are jointly determined by modifiable health beliefs (perceived benefits, self-efficacy) and socioeconomic resources (education, income). Public health interventions should integrate benefit-focused education, self-efficacy enhancement programs, and structural strategies to reduce access barriers for disadvantaged subgroups, particularly those with lower education and income levels.

## 1 Introduction

**Figure 1:**
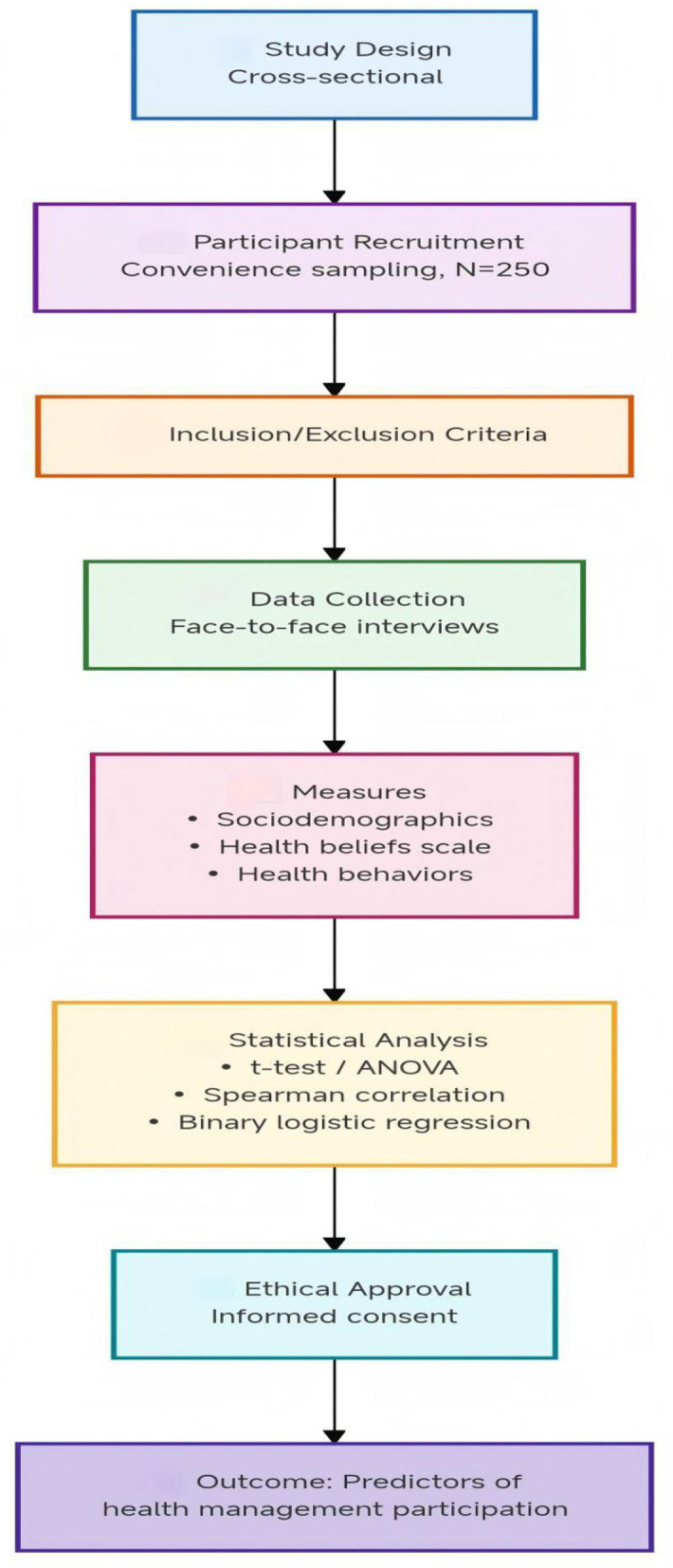
Summary chart of arguments in the introduction part.

The global population is aging at an unprecedented rate, presenting significant challenges to public health systems worldwide (1). China is experiencing a rapid demographic shift, with its population aged 60 years and above reaching 21.1% in 2025 (2). In Nanning, by the end of 2024, the aging rate of residents over 60 was 18.59% (42). Although the degree of aging in Nanning is slightly below the national average, its characteristics of a “large base and many older adult of advanced age” pose urgent demands on local health management work. This trend is particularly evident in urban centers such as Nanning, the capital of Guangxi Zhuang Autonomous Region, posing specific challenges for chronic disease management and healthcare delivery (3). Effective health management among older adults—including regular health checks, adherence to medical advice, and active disease prevention—is crucial for controlling disease incidence, improving quality of life, and optimizing the allocation of medical resources (4). A flowchart detailing the cross-sectional study process is presented in Figure 1.

The “Healthy China 2030” initiative explicitly emphasizes strengthening health management services for older adults (5). However, despite policy efforts, the participation rate in community-based health management programs remains suboptimal. Previous studies have identified multiple barriers among Chinese older adults, including traditional health beliefs, limited health literacy, financial constraints, and low trust in community healthcare providers (6–8). These barriers suggest that improving service coverage alone is insufficient; a deeper understanding of the psychological and social determinants that shape older adults' health-related decision-making is urgently needed. In particular, HBM provides a robust framework for examining how individual perceptions influence health behaviors, yet its application to general health management among urban community-dwelling older adult in specific regional contexts—such as Nanning City—remains limited. Therefore, the present study aims to identify the key psychological and social determinants of health management adherence among Chinese older adults, thereby addressing this critical gap and informing theory-based strategies to improve participation.

The HBM, originally developed by Rosenstock (9, 10) in the 1970s, remains one of the most widely used theoretical frameworks for explaining and predicting health-related behaviors. The model posits that an individual's likelihood of engaging in a health behavior is influenced by five core perceptual dimensions: perceived susceptibility (belief about the risk of contracting a condition), perceived severity (belief about the seriousness of the condition), perceived benefits (belief about the effectiveness of the recommended action), perceived barriers (belief about the costs or obstacles of taking action), and self-efficacy (confidence in one's ability to successfully perform the action) (11, 12). According to the model, individuals are most likely to adopt a health behavior when they perceive themselves as susceptible to a serious condition, believe the recommended action is beneficial and outweighs the barriers, and feel confident in their ability to execute it (13).

The HBM has been extensively applied to investigate health behaviors among older adult populations, including cancer screening, medication adherence, diabetes self-management, and preventive service utilization (14, 15). A substantial body of evidence consistently identifies perceived benefits and self-efficacy as the strongest positive predictors of behavior, while perceived barriers act as significant deterrents (16, 17). Moreover, socioeconomic factors such as educational attainment and income level interact with these health beliefs, shaping behavioral outcomes by influencing access to health information, literacy, and financial resources (18, 19). Higher educational attainment is associated with better health literacy, which enhances perceived benefits and self-efficacy, whereas financial constraints amplify perceived barriers and reduce the likelihood of action (20, 21).

In the context of chronic disease management, the HBM provides a useful analytical lens. While a diagnosis of chronic illness may heighten an individual's perceived susceptibility and severity, this heightened threat perception does not automatically translate into consistent self-management behaviors (22). The role of self-efficacy becomes paramount, as managing a chronic condition requires ongoing confidence, problem-solving skills, and behavioral persistence (23). Social support—particularly from family members—has also been identified as a critical cue to action that can reduce perceived barriers and enhance self-efficacy (24, 25).

Despite the extensive application of the HBM globally, research examining its utility in understanding health management behaviors among community-dwelling older adults in specific regional contexts within China—such as Nanning City—remains limited. Most existing studies have focused on clinical populations with specific diseases or have relied on generalized national samples that overlook local socioeconomic and cultural variations (26, 27). Furthermore, few studies have systematically examined how the interplay between HBM constructs and socioeconomic factors influences the “knowledge-attitude-practice” pathway among older adults in urban community settings in China.

Therefore, this study aims to fill this research gap by addressing the following objectives:

(1) To assess the current status of health management cognition, behaviors, and health beliefs among older adults in a representative urban community in Nanning;
(2) To examine the bivariate relationships between HBM dimensions and health management behavior;
(3) To identify the independent effects of health beliefs and sociodemographic characteristics on participation in health management services using multivariate analysis.

## 2 Methods

### 2.1 Study design and participants

**Figure 2:**
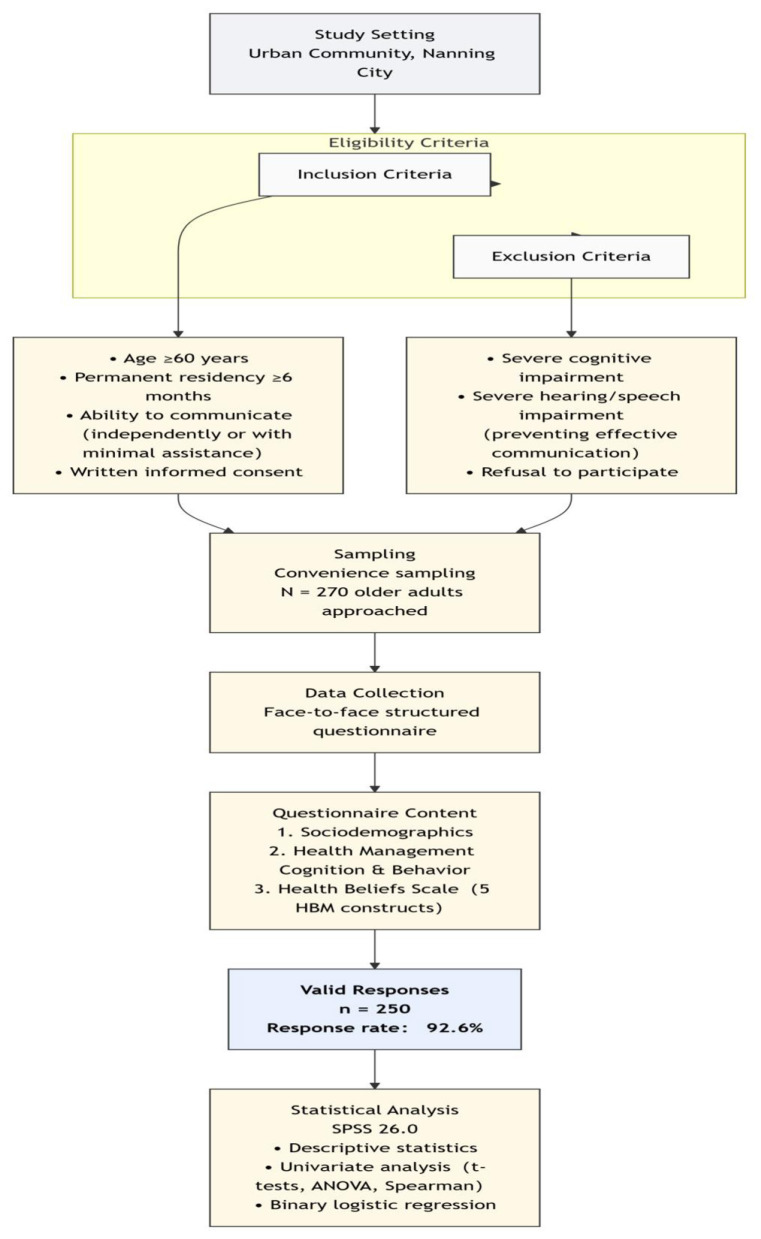
Flow chart of steps for research methodology.

This descriptive cross-sectional study was conducted between March and June 2024 in an urban community in Nanning City, Guangxi Zhuang Autonomous Region, China. The target population comprised permanent residents aged 60 years or older who had resided in the community for at least 6 months This time frame was selected to coincide with the annual community health promotion campaign organized by the Nanning Municipal Health Commission, which facilitated participant recruitment and ensured a representative cross-section of community-dwelling older adults. The sampling and analysis workflow is illustrated in Figure 2.

Inclusion criteria: (1) age ≥60 years; (2) permanent residency in the selected community; (3) ability to communicate and complete the survey independently or with minimal assistance; (4) provision of written informed consent.

Exclusion criteria: (1) severe cognitive impairment (e.g., dementia or mental disorders) that precluded understanding of the questionnaire; (2) severe hearing or speech impairment preventing effective communication; (3) refusal to participate; and (4) participants with chronic physical conditions (e.g., hypertension, diabetes) were not excluded, as the study aimed to capture health management behaviors across the full spectrum of health status among community-dwelling older adult.

Sample size estimation: following Kendall's sample size estimation principle for multivariate analysis, a minimum of 10–20 cases per independent variable is recommended. With 11 independent variables included in the logistic regression model, the required sample size was estimated at 110–220 participants. To account for an anticipated 20% invalid response rate, a target sample of 270 participants was set.

A convenience sampling strategy was employed. With the assistance of community health workers and neighborhood committee staff, 270 older adults were approached. After excluding 20 invalid questionnaires due to incomplete responses or logical inconsistencies, 250 valid questionnaires were retained, yielding an effective response rate of 92.6%.

Ethical considerations: all participants provided written informed consent prior to data collection. Participants were assured of their right to withdraw at any time without consequences. Anonymity and confidentiality were strictly maintained throughout the research process. The data collection and research procedures described in this paper were conducted in accordance with the Ethical Review standards of Guilin Medical University. This research is supported by the Middle-aged and Young Teachers' Basic Research Capacity Enhancement Project (Grant No. 2024KY0493). The ethical compliance for this project was formally reviewed and approved during the grant application process under the aforementioned funding number.

### 2.2 Measures

A structured questionnaire was developed based on an extensive literature review and expert consultation. Data were collected through face-to-face interviews conducted by trained investigators (two public health graduate students). The questionnaire comprised three sections:

#### 2.2.1 Sociodemographic characteristics

Sociodemographic variables included: gender (male, female); age (categorized as 60–65, 66–70, 71–75, ≥76 years); educational level (primary school or below, middle school, high school/vocational school, college or above); monthly personal income (< 1,000, 1,001–2,000, 2,001–3,000, >3,000 RMB); chronic disease status (presence of at least one physician-diagnosed chronic condition such as hypertension, diabetes, or cardiovascular disease: yes/no); living situation (living alone, living with family, residing in a nursing facility).

#### 2.2.2 Health management cognition and behaviors

In this study, “health management behavior” was operationalized as having ever received any of the following services provided by the community health center or other healthcare institutions: regular health examinations (at least once in the past 2 years), chronic disease follow-up visits, or participation in organized health education activities. This definition aligns with the core components of China's basic public health services for the older adult but also encompasses self-initiated health promotion activities.

This section assessed: access to health management knowledge (yes/no; primary sources); awareness of the importance of health management (5-point Likert scale: 1 = not important to 5 = very important); knowledge of chronic disease risk factors (fully aware, partially aware, not aware); prior receipt of health management services (yes/no); frequency of regular physical examinations (every 6 months, once a year, occasionally, never); reasons for non-participation (multiple choice).

#### 2.2.3 Health Belief Scale

The Health Belief Scale was adapted from the classical HBM scales developed by Champion and Skinner (11) and Janz and Becker (13), with cultural and contextual modifications. The adaptation process involved: (1) forward translation from English to Chinese by two independent bilingual researchers; (2) back-translation by a third researcher; (3) expert review by a panel of three public health and gerontology experts; and (4) pilot testing among 30 older adults to assess clarity, comprehensibility, and cultural appropriateness.

The final scale comprised 10 items measuring five HBM dimensions (two items per dimension):

- Perceived susceptibility: belief about the likelihood of developing health problems (e.g., “I believe I am at risk of developing chronic diseases”)
- Perceived severity: belief about the seriousness of health problems (e.g., “I believe chronic diseases seriously affect quality of life”)
- Perceived benefits: belief about the effectiveness of health management actions (e.g., “Regular health checks can help detect diseases early”)
- Perceived barriers: belief about obstacles to engaging in health management (e.g., “Health management services are too expensive”)
- Self-efficacy: confidence in the ability to perform health management actions (e.g., “I am confident I can adhere to health advice even when it is inconvenient”)

All items were rated on a 5-point Likert scale (1 = strongly disagree, 2 = disagree, 3 = neutral, 4 = agree, 5 = strongly agree). Dimension scores were calculated as the mean of the two constituent items. Higher scores indicate stronger beliefs in that dimension, except for perceived barriers, where higher scores indicate greater perceived obstacles. Dimension scores were calculated as the mean of the two constituent items to retain the original 1–5 metric, facilitating intuitive interpretation and comparison across dimensions. This approach is consistent with standard practices in HBM research (11, 13).

Reliability and validity:

- Internal consistency: the total scale demonstrated acceptable reliability (Cronbach's α = 0.81). Dimension-specific Cronbach's α values were: perceived susceptibility (0.78), perceived severity (0.80), perceived benefits (0.76), perceived barriers (0.79), and self-efficacy (0.85).
- Construct validity: Confirmatory factor analysis (*CFA*) was conducted using AMOS 26.0. The five-factor model demonstrated good fit: χ^2^*/*df = 2.31, *RMSEA* = 0.05 (*90%* CI: 0.03–0.07), *CFI* = 0.94, *SRMR* = 0.04, all exceeding recommended thresholds. Factor loadings ranged from 0.62 to 0.88 (all *P* < 0.001).

A limitation of the current HBM measurement is the exclusion of the “cues to action” dimension, which is an integral component of the original model. Although items assessing information sources and social support (e.g., living with family) were included in the sociodemographic section, a dedicated scale for cues to action was not administered. This omission may reduce the explanatory completeness of the model, and future studies should incorporate validated measures of internal and external cues to action.

### 2.3 Data collection procedure

Investigators underwent a standardized training session covering interview techniques, ethical conduct, and questionnaire administration. Interviews were conducted in private rooms at the community health service center or participants' homes, according to participant preference. Each interview lasted approximately 20–25 min. For participants with visual difficulties or low literacy, investigators read the questions aloud and recorded responses.

Quality control measures:

- All completed questionnaires were checked on-site for completeness.
- Data were double-entered independently by two researchers using EpiData 3.1 and cross-validated.
- Logical consistency checks were performed to identify and correct entry errors.
- Questionnaires with >20% missing data or obvious response patterns (e.g., all identical answers) were excluded.

### 2.4 Statistical analysis

Statistical analyses were performed using SPSS version 26.0 (IBM Corp., Armonk, NY, USA). A two-tailed *P-value* < 0.05 was considered statistically significant.

Descriptive statistics: continuous variables were presented as mean ± standard deviation (SD); categorical variables were presented as frequencies and percentages.

Bivariate analysis:

- Independent *t*-tests and one-way ANOVA were used to compare health belief scores across sociodemographic subgroups.
- Spearman's rank correlation was employed to examine associations between health belief dimensions and health management needs/behaviors.

Multivariate analysis:

Binary logistic regression (Enter method) was conducted to identify independent predictors of health management behavior (dichotomized dependent variable: 1 = has received health management services, 0 = never received). Independent variables included: sociodemographic characteristics (gender, age, education, income, chronic disease status, living situation) and all five health belief dimensions. Health belief dimension scores were entered as continuous variables to preserve statistical power and avoid arbitrary categorization.

Model diagnostics:

- Multicollinearity was assessed using variance inflation factors (*VIF*); all *VIF* values were < 2.0, indicating no serious multicollinearity.
- Model fit was evaluated using the Hosmer–Lemeshow goodness-of-fit test.
- Overall predictive accuracy was assessed by the percentage of correctly classified cases.
- Adjusted odds ratios (*ORs*) and 95% confidence intervals (*CIs*) were reported.

## 3 Results

### 3.1 Participant characteristics

**Figure 3:**
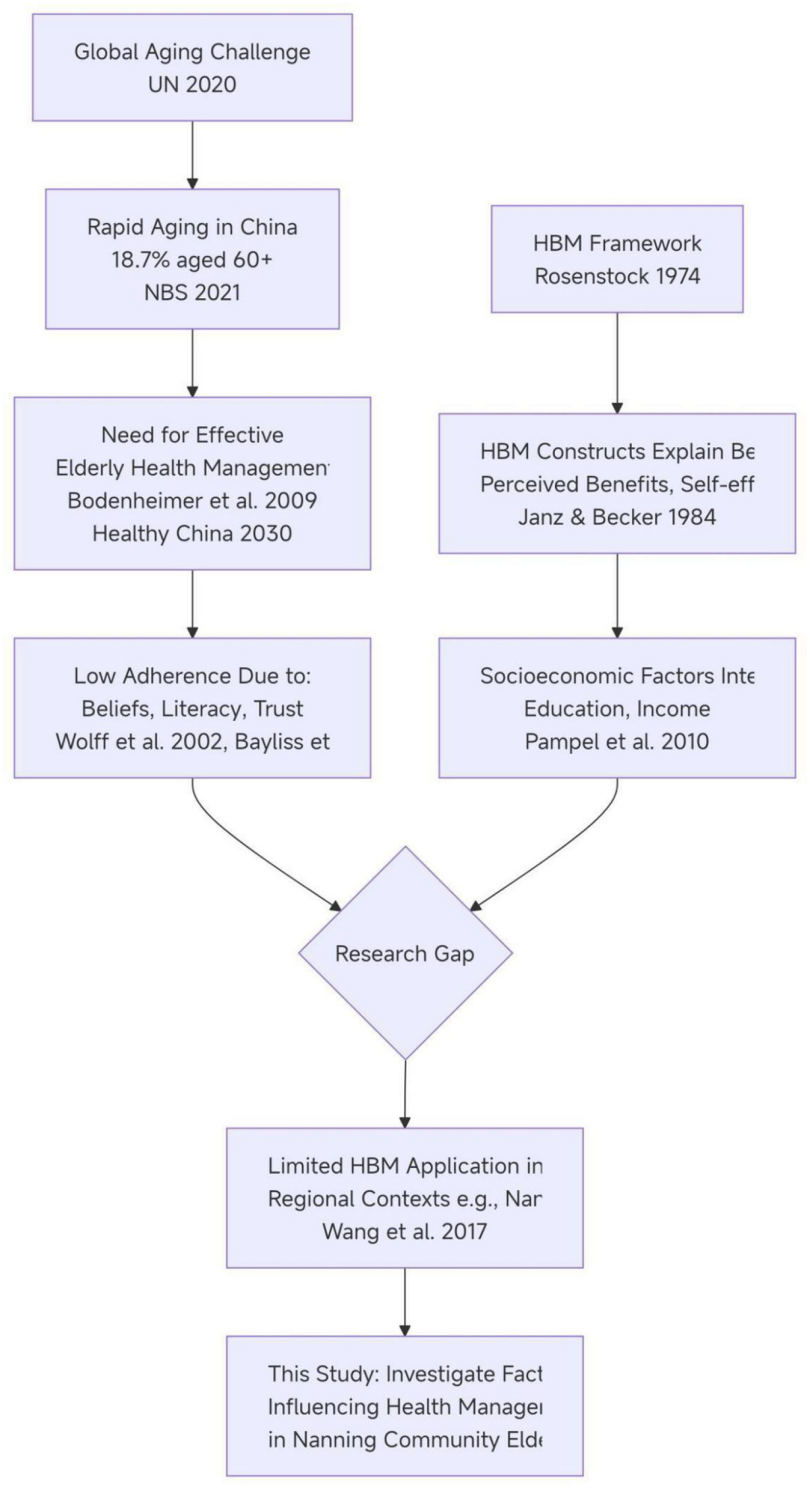
Logical relationship diagram of research methods.

A total of 250 older adults participated in this study. The mean age was 70.1 ± 6.8 years (range: 60–92 years). Female participants accounted for 51.2% (*n* = 128). Regarding educational attainment, 32.0% (*n* = 80) had completed primary school or below, 24.8% (*n* = 62) middle school, 28.4% (*n* = 71) high school or vocational school, and 14.8% (*n* = 37) held a college degree or above. The most frequently reported monthly income bracket was 2,001–3,000 RMB (32.0%, *n* = 80). Over half of the participants (58.4%, *n* = 146) reported having at least one physician-diagnosed chronic disease. Regarding living arrangements, 64.4% (*n* = 156) lived with family, 28.0% (*n* = 70) lived alone, and 9.6% (*n* = 24) resided in nursing facilities. Detailed sociodemographic characteristics of the 250 participants are presented in Table 1. The rationale for this study is outlined in Figure 3.

**Table 1:** Sociodemographic characteristics of participants (*N* = 250).

Variable	Category	Number of people	Percentage (%)
Gender	Male	122	48.8
	Female	128	51.2
Age	60–65 years	66	26.4
	66–70 years	72	28.8
	71–75 years	60	24.0
	76 years and above	52	20.8
Education level	Primary school and below	80	32.0
	Middle school	62	24.8
	High school/Technical secondary	71	28.4
	College and above	37	14.8
Monthly income (RMB)	< 1,000	46	18.4
	1,001–2,000	64	25.6
	2,001–3,000	80	32.0
	>3,000	60	24.0
Whether suffering from chronic disease	Yes	146	58.4
	No	104	41.6
Living situation	Living alone	70	28.0
	Living with family	156	64.4
	Nursing facility	24	9.6

### 3.2 Health management cognition and behaviors

Table 2 presents the distribution of health management cognition (knowledge access channels, awareness of importance, understanding of risk factors) and behaviors (service utilization, physical examination frequency) among community-dwelling older adults in Nanning, China.

**Table 2:** Health management cognition and behaviors among participants (*N* = 250)

Category	Specific project/Channel	Number of people	Percentage (%)	Rank
Sources of health management knowledge^a^	Community outreach	114	45.6	1
	Healthcare workers	82	32.8	2
	TV/Radio	56	22.4	3
	Recommendations from family and friends	48	19.2	4
	Online platforms	34	13.6	5
	Books/Newspapers	26	10.4	6
Health management cognition	Access to health management knowledge—Yes	198	79.2	
	Access to health management knowledge—No	52	20.8	
	Awareness of the importance of health management—Very important	128	51.2	
	Awareness of the importance of health management—Important	84	33.6	
	Awareness of the importance of health management—Neutral	26	10.4	
	Awareness of the importance of health management—Not important	8	3.2	
	Knowledge of chronic disease risk factors—Fully Aware	32	12.8	
	Knowledge of chronic disease risk factors—Partially aware	160	64	
	Knowledge of chronic disease risk factors—Not aware	58	23.2	
Health management behaviors	Received health management services—Yes	136	54.4	
	Received health management services—No	114	45.6	
	Regular physical exam frequency—Once every 6 months	52	20.8	
	Regular physical exam frequency—Once a year	78	31.2	
	Regular physical exam frequency—Occasionally	84	33.6	
	Regular physical exam frequency—Never	36	14.4	

^a^Percentages calculated based on total sample (N = 250) due to multiple responses allowed.

Access to health information: the majority of participants (79.2%, *n* = 198) reported having access to health management knowledge. The primary sources of health information were community publicity (45.6%, *n* = 114) and healthcare workers (32.8%, *n* = 82). Other sources included television/radio (22.4%, *n* = 56), recommendations from family and friends (19.2%, *n* = 48), online platforms (13.6%, *n* = 34), and books/newspapers (10.4%, *n* = 26; Table 2).

Awareness and knowledge: although 84.8% (*n* = 212) of participants considered health management to be important or very important, only 12.8% (*n* = 32) could fully list common chronic disease risk factors. Nearly two-thirds (64.0%, *n* = 160) had partial knowledge, and 23.2% (*n* = 58) were unaware of any risk factors (Table 2).

Health management behaviors: only 54.4% (*n* = 136) of participants reported having ever received any form of health management services. Regular physical examination attendance was suboptimal: 20.8% (*n* = 52) underwent examinations every 6 months, 31.2% (*n* = 78) once a year, 33.6% (*n* = 84) occasionally, and 14.4% (*n* = 36) never (Table 2). Among those who had never received services, the most frequently cited reasons were “lack of access to health management knowledge” (20.8%), “high cost” (16.4%), “complex procedures” (15.6%), and “no perceived need” (14.0%).

### 3.3 Descriptive statistics of health belief constructs

Overall health belief scores: the overall mean score for health beliefs was 3.42 ± 0.68. Among the five dimensions, perceived benefits scored highest (3.85 ± 0.72), followed by perceived severity (3.64 ± 0.69), perceived susceptibility (3.52 ± 0.75), self-efficacy (3.12 ± 0.81), and perceived barriers (2.97 ± 0.88; Table 3). This pattern indicates that participants generally recognized the benefits of health management but reported relatively low confidence in their ability to perform health management behaviors and moderate levels of perceived obstacles.

**Table 3:** Descriptive statistics of health belief dimensions (*N* = 250).

Dimension	Average score	Ranking
Perceived severity	3.64 ± 0.69	2
Perceived benefits	3.85 ± 0.72	1
Perceived barriers	2.97 ± 0.88	5
Self-efficacy	3.12 ± 0.81	4
Perceived susceptibility	3.52 ± 0.75	3
Overall average score	3.42 ± 0.68	

Table 3 displays the mean scores and rankings of the five HBM dimensions among community-dwelling older adults in Nanning, with perceived benefits scoring the highest and perceived barriers the lowest, and an overall mean health belief score of 3.42 ± 0.68.

### 3.4 Differences in health beliefs by sociodemographic characteristics

Education: significant differences were observed in perceived benefits (*F* = 2.76, *P* = 0.04) and self-efficacy (*F* = 2.34, *P* = 0.07) across educational levels. Participants with a college degree or above reported substantially higher perceived benefits (4.12 ± 0.65) and self-efficacy (3.45 ± 0.72) compared to those with primary education or below (3.72 ± 0.75 and 2.98 ± 0.85, respectively).

Chronic disease status: participants with at least one chronic disease reported significantly higher perceived severity (3.89 ± 0.71 vs. 3.32 ± 0.68; *t* = 4.52, *P* < 0.01) and perceived susceptibility (3.62 ± 0.73 vs. 3.38 ± 0.77; *t* = 2.16, *P* = 0.03) compared to those without chronic conditions.

Living situation: living alone was associated with significantly higher perceived barriers (3.25 ± 0.82) compared to living with family (2.81 ± 0.76) or in nursing facilities (2.90 ± 0.80; *F* = 3.78, *P* = 0.02).

Gender and age: no statistically significant differences were found across gender or age groups for any health belief dimension (all *P* > 0.05). Detailed comparisons are presented in Table 4.

**Table 4:** Comparison of health belief scores by sociodemographic characteristics (*N* = 250).

Variable	Perceived severity	Perceived benefits	Perceived barriers	Self efficacy	Perceived susceptibility	Overall average score
Gender						
Male	3.62 ± 0.71	3.82 ± 0.75	3.01 ± 0.91	3.08 ± 0.83	3.50 ± 0.77	3.40 ± 0.70
Female	3.66 ± 0.67	3.88 ± 0.69	2.93 ± 0.85	3.16 ± 0.79	3.54 ± 0.73	3.44 ± 0.66
*T/P*	0.37/0.71	0.56/0.58	0.62/0.54	0.67/0.50	0.34/0.73	0.42/0.67
Age						
60–65 years old	3.58 ± 0.72	3.80 ± 0.78	2.92 ± 0.90	3.05 ± 0.85	3.48 ± 0.79	3.38 ± 0.72
66–70 years old	3.66 ± 0.68	3.86 ± 0.70	2.98 ± 0.86	3.10 ± 0.80	3.54 ± 0.74	3.42 ± 0.68
71–75 years old	3.68 ± 0.66	3.88 ± 0.68	3.02 ± 0.84	3.15 ± 0.78	3.56 ± 0.72	3.45 ± 0.64
76 years old and above	3.62 ± 0.69	3.82 ± 0.74	2.90 ± 0.92	3.08 ± 0.82	3.46 ± 0.76	3.39 ± 0.70
*F/P*	0.24/0.87	0.18/0.91	0.21/0.89	0.16/0.93	0.14/0.94	0.15/0.92
Education level						
Elementary or below	3.54 ± 0.70	3.72 ± 0.75^*^	3.05 ± 0.90	2.98 ± 0.85	3.44 ± 0.77	3.32 ± 0.72
Middle school	3.62 ± 0.68	3.80 ± 0.72	2.96 ± 0.86	3.06 ± 0.82	3.50 ± 0.74	3.40 ± 0.68
High school/Vocational school	3.68 ± 0.66	3.88 ± 0.68	2.92 ± 0.84	3.14 ± 0.78	3.56 ± 0.72	3.44 ± 0.64
College and above	3.82 ± 0.62	4.12 ± 0.65	2.80 ± 0.80	3.45 ± 0.72	3.68 ± 0.68	3.58 ± 0.60
*F/P*	1.84/0.14	2.76/0.04	0.78/0.51	2.34/0.07	1.48/0.22	2.02/0.11
Whether suffering from chronic disease						
Yes	3.89 ± 0.71^**^	3.9 2± 0.70	2.94 ± 0.85	3.10 ± 0.79	3.62 ± 0.73	3.49 ± 0.66
No	3.32 ± 0.68^**^	3.76 ± 0.74	3.00 ± 0.90	3.14 ± 0.83	3.38 ± 0.77	3.32 ± 0.70
*T/P*	4.52/ < 0.01	1.38/0.17	0.42/0.67	0.34/0.73	2.16/0.03	1.78/0.08
Living situation						
Living alone	3.6 0± 0.70	3.80 ± 0.75	3.25 ± 0.82^*^	3.06 ± 0.84	3.48 ± 0.76	3.39 ± 0.70
Living with family	3.66 ± 0.68	3.88 ± 0.69	2.81 ± 0.76^*^	3.14 ± 0.79	3.54 ± 0.74	3.4 4± 0.66
Nursing facility	3.62 ± 0.66	3.82 ± 0.72	2.90 ± 0.80^*^	3.08 ± 0.80	3.50 ± 0.72	3.40 ± 0.64
*F/P*	0.23/0.88	0.21/0.89	3.78/0.02	0.22/0.80	0.16/0.85	0.20/0.82

Bolded values for education level (*P*=0.04 for perceived benefits) and chronic disease status (*P* < 0.01 for perceived severity).

Table 4 compares health belief dimension scores across sociodemographic subgroups (gender, age, education level, chronic disease status, and living situation), revealing that education level is significantly associated with perceived benefits, chronic disease status with perceived severity and susceptibility, and living situation with perceived barriers.

### 3.5 Bivariate associations between health beliefs and health management behaviors

Correlation analysis: Spearman's rank correlation revealed that health management behavior (ever received services) was positively correlated with perceived benefits (*r* = 0.36, *P* < 0.01) and self-efficacy (*r* = 0.28, *P* < 0.01). A weak negative correlation was observed with perceived barriers (*r* = −0.22, *P* = 0.15), although this did not reach statistical significance. Perceived susceptibility and perceived severity were not significantly correlated with behavior (*P* > 0.05).

Cognition-behavior linkage: participants who had access to health management knowledge reported significantly higher self-efficacy (3.45 ± 0.73 vs. 2.68 ± 0.79; *t* = 4.52, *P* < 0.01) and lower perceived barriers (2.90 ± 0.84 vs. 3.20 ± 0.92; *t* = −2.16, *P* = 0.03) compared to those without access. Furthermore, participants who fully understood chronic disease risk factors demonstrated higher perceived susceptibility (3.68 ± 0.68) and self-efficacy (3.45 ± 0.72), although the latter difference was not statistically significant (*P* = 0.12). Detailed comparisons are presented in Table 5.

**Table 5:** Association between health management cognition and health beliefs (*N* = 250).

Cognitive dimension	Health belief dimension	Mean scores (comparison groups)	Statistical test used	Statistical test results (*T*/*F* values, *P* values)
Knowledge acquisition methods	Self-efficacy	3.45 ± 0.73 vs. 2.68 ± 0.79	Independent *t*-test	*T* = 4.52, ***P*** **<** **0.01**
	Perceived barriers	2.90 ± 0.84 vs. 3.20 ± 0.92	Independent *t*-test	*T* = −2.16, *P* = 0.03
Awareness of the importance of health management	Perceived benefits	Very important: 4.12 ± 0.65	One-way ANOVA	*F* = 4.52, ***P*** **< 0.01**
Understanding of chronic disease risk factors	Perceived susceptibility	Got it: 3.68 ± 0.68	Spearman correlation	*r* = 0.36, *P* < 0.01
	Self-efficacy	Got it: 3.45 ± 0.72	One-way ANOVA	*F* = 2.16, *P* = 0.12
	Overall average score	Very important: 3.58 ± 0.60	Spearman correlation	*r* = 0.18, *P* = 0.06

Bolded values for knowledge acquisition (*P* < 0.01 for self-efficacy) and awareness of importance (*P* < 0.01 for perceived benefits).

Table 5 demonstrates that better health management cognition—including access to knowledge, awareness of importance, and understanding of risk factors—is significantly associated with stronger self-efficacy, lower perceived barriers, higher perceived benefits, and greater perceived susceptibility among older adults.

#### 3.5.1 Stratified analysis of socioeconomic status and health management participation

A supplementary stratified analysis was performed to examine potential dose-response relationships. Health management participation rates increased progressively with educational level: 42.5% (primary school or below), 51.6% (middle school), 58.2% (high school/vocational), and 67.6% (college or above). The Cochran-Armitage test for trend was significant (χ^2^ = 6.42, *P* = 0.011). Similarly, participation rates increased across income brackets: 39.1% (< 1,000 RMB), 50.0% (1,001–2,000 RMB), 58.8% (2,001–3,000 RMB), and 65.0% (>3,000 RMB; χ^2^ trend = 7.18, *P* = 0.007). These patterns suggest a dose-response relationship, though the gradient is moderate and should be interpreted with caution given the limited sample size within each stratum.

### 3.6 Predictors of health management behavior: binary logistic regression

A binary logistic regression model was constructed to identify independent predictors of health management behavior (receipt of services). The model included all sociodemographic variables and the five health belief dimensions as continuous predictors. The Hosmer–Lemeshow test indicated good model fit (χ^2^ = 8.64, df = 8, *P* = 0.37). The overall classification accuracy was 78.2%, with a Nagelkerke *R*^2^ of 0.34.

Significant independent predictors:

After controlling for all covariates, five variables emerged as statistically significant predictors:

1. Educational level (OR = 1.38, 95% CI: 1.01–1.90, *P* = 0.04): higher education was associated with 38% greater odds of engaging in health management.
2. Monthly income (OR = 1.32, 95% CI: 1.01–1.72, *P* = 0.04): higher income increased the odds by 32%.
3. Chronic disease status *(*OR = 1.52, 95% CI*:* 1.03–2.23, *P* = 0.03): having at least one chronic condition nearly doubled the odds of participation.
4. Perceived benefits (OR = 1.62, 95% CI: 1.09–2.40, *P* = 0.02): each one-point increase in perceived benefits score increased the odds by 62%.
5. Self-efficacy (OR = 1.68, 95% CI: 1.10–2.56, *P* = 0.02): each one-point increase in self-efficacy score increased the odds by 68%.

Non-significant factors: gender, age, living situation, perceived susceptibility, perceived severity, and perceived barriers were not independently associated with health management behavior in the multivariate model (all *P* > 0.05). Perceived barriers showed a trend toward significance (OR = 0.69, 95% CI: 0.47–1.02, *P* = 0.07) in the expected negative direction.

Multicollinearity diagnostics: all variance inflation factors (VIF) were below 2.0, indicating no serious multicollinearity concerns.

Full regression results are presented in Table 6.

**Table 6:** Binary logistic regression analysis of factors influencing health management behavior (*N* = 250).

Influencing factors	Partial regression coefficient	Standard error	Wald	Degrees of freedom	*P*	OR	95% confidence interval of OR	
							Lower limit	Upper limit
Gender	−0.12	0.28	0.18	1	0.67	0.89	0.52	1.53
Age	0.08	0.24	0.11	1	0.74	1.08	0.68	1.72
Educational level	0.32	0.16	4	1	0.04	1.38	1.01	1.9
Monthly income	0.28	0.14	4		0.04	1.32	1.01	1.72
Whether suffering from chronic disease	0.42	0.2	4.41	1	0.03	1.52	1.03	2.23
Living situation	−0.26	0.22	1.44		0.23	0.77	0.5	1.19
Perceived severity	0.24	0.18	1.78	1	0.18	1.27	0.89	1.82
Perceived benefits	0.48	0.2	5.76		0.02	1.62	1.09	2.4
Perceived barriers	−0.36	0.2	3.24	1	0.07	0.69	0.47	1.02
Self-efficacy	0.52	0.22	5.52	1	0.02	1.68	1.1	2.56
Perceived susceptibility	0.22	0.18	1.44		0.23	1.25	0.88	1.78
Constant	−1.24	0.56	4.96	1	0.03	0.29		

Dependent variable: 1 = has received health management services (n = 136), 0 = never received (n = 114).

B, unstandardized coefficient; S.E., standard error; OR, odds ratio; CI, confidence interval.

Hosmer–Lemeshow test: χ^2^ = 8.64, df = 8, P = 0.37. Nagelkerke R^2^ = 0.34. Overall classification accuracy: 78.2%.

## 4 Discussion

This study applied the HBM to investigate the factors influencing health management behaviors among community-dwelling older adults in Nanning, China. Our findings demonstrate that participation in health management is jointly determined by modifiable health beliefs—particularly perceived benefits and self-efficacy—and socioeconomic resources such as education and income. These results provide empirical evidence to inform targeted interventions aimed at improving health management adherence in this population. We first discuss the central psychological drivers of behavior, then address non-significant findings, and finally situate our results within the broader literature.

### 4.1 The central role of perceived benefits and self-efficacy

Consistent with the theoretical propositions of the HBM (13) and a substantial body of empirical research (14, 16), perceived benefits and self-efficacy emerged as the strongest independent psychological predictors of health management behavior. Participants who strongly believed in the effectiveness of health management actions were 62% more likely to engage in them, while those with greater confidence in their ability to perform such actions were 68% more likely to participate. These effect sizes are clinically meaningful and highlight the critical importance of cognitive appraisal processes in behavioral decision-making among older adults.

The predominance of perceived benefits aligns with previous studies conducted among older populations in China and internationally (28, 29). When older adults clearly understand that regular health checks can detect diseases early, that adherence to medical advice prevents complications, and that health management improves quality of life, they are substantially more motivated to act. This finding underscores the need for health education interventions that explicitly communicate the tangible benefits of health management using simple, culturally appropriate, and personally relevant messaging. For instance, community health workers in Nanning could use local dialect and relatable scenarios (e.g., linking regular blood pressure monitoring to the ability to continue caring for grandchildren or participating in popular local square dancing) to make benefits more vivid and personally meaningful. Additionally, leveraging trusted community channels such as senior activity centers and neighborhood committee meetings for benefit-focused education could enhance message reach and receptiveness.

The finding that self-efficacy was the single strongest predictor (OR = 1.68) corroborates Bandura's social cognitive theory (12) and extensive research on chronic disease self-management (23, 30). Self-efficacy is not merely confidence; it reflects an individual's perceived capability to organize and execute courses of action despite obstacles (11). Among older adults, low self-efficacy may stem from age-related physical decline, lack of prior experience with health management, insufficient health literacy, or negative past experiences with healthcare systems (31). Our finding that participants with higher education and those with access to health information reported significantly higher self-efficacy suggests that knowledge acquisition and mastery experiences are critical pathways for confidence-building.

Based on the local community health service context, specific, culturally tailored recommendations are provided to enhance perceived benefits and self-efficacy among older adults in Nanning:

1. Benefit-focused health talks leveraging existing “Health Lecture Hall” programs: Enhance these talks with video testimonials from local older adults who have successfully managed chronic conditions, filmed in familiar settings like Litchi Park using local dialect (Nanning Pinghua or Cantonese). For example: “After 3 months of regular monitoring, I can now walk to the senior canteen without getting winded.” Integrate these talks into existing senior gatherings, such as Neighborhood Committee Meetings or morning square dancing at Nanhu Park, to maximize attendance without.
2. Self-efficacy enhancement through low-literacy goal-setting tools: develop a “Daily Health Goal Checklist” booklet with large-font text (≥16 pt), pictograms, color-coded sections, and a simple checkbox format, displaying only 3–4 goals per page. Pilot this tool at Mingxiu South Community, where the senior canteen serves 500+ meals daily. During routine follow-ups, family doctor teams spend 3–5 min reviewing progress. To sustain engagement, offer a small incentive (e.g., free meal voucher) after 30 days of completed tracking.
3. Community-based skill stations modeled on existing “Smart Health Cabinets”: establish “Health Skill Experience Corners” within existing facilities like Xiushan Community, where older adults practice using medication reminder boxes and home blood pressure monitors under supervision of volunteers from the “Qihuang Volunteer Program.” For homebound older adults, use a train-the-trainer model with 15–20 min home visits to demonstrate device use, using locally purchased devices.

Future perspectives: interventions should move beyond didactic education to incorporate skills-building components, such as demonstration sessions, practice opportunities, goal-setting exercises, and peer modeling. Chronic disease self-management programs (CDSMP) developed by Lorig et al. (23, 32) offer evidence-based templates that could be adapted for community-dwelling older adults in urban China.

### 4.2 In health management participation

While health beliefs are modifiable at the individual level, socioeconomic resources shape the context within which these beliefs operate. This study identified significant socioeconomic disparities in health management behavior. Participants with higher educational attainment and higher monthly income were significantly more likely to engage in health management, even after accounting for health beliefs. The stratified analysis further revealed a stepwise increase in participation across educational and income tiers, suggesting a modest dose-response pattern consistent with a socioeconomic disparities. However, given the relatively small sample size within individual strata, these findings should be viewed as preliminary evidence rather than definitive confirmation.

Education likely exerts its influence through multiple mechanisms. Higher education is associated with better health literacy—the capacity to obtain, process, and understand basic health information needed to make appropriate health decisions (21). Health literacy enables individuals to recognize the importance of prevention, navigate complex healthcare systems, and communicate effectively with providers. Our observation that college-educated participants scored significantly higher on perceived benefits and self-efficacy supports this mechanism. Furthermore, education shapes cognitive schemas about health and illness, fostering a future-oriented, proactive orientation toward health maintenance (33).

Income affects health management participation through material constraints. Despite China's substantial progress in expanding primary healthcare coverage, out-of-pocket costs for certain preventive services, transportation expenses, and opportunity costs (e.g., time off work for family caregivers) remain barriers for low-income older adults (34). Our finding that the most commonly cited reason for non-participation was “lack of access to knowledge” (a cognitive barrier) rather than cost alone suggests that financial barriers may be compounded by informational barriers in this population.

Chronic disease status emerged as a powerful facilitator of health management behavior, consistent with the HBM's conceptualization of personal experience as a cue to action (13). Individuals living with chronic conditions have direct experiential knowledge of illness and have typically received repeated recommendations for disease management from healthcare providers. However, it is noteworthy that nearly half (45.6%) of participants with chronic diseases in our sample had never received formal health management services, indicating a substantial service gap. This finding echoes previous research demonstrating that disease diagnosis alone is insufficient to guarantee sustained self-management behaviors (22, 35). Nevertheless, because our measure of health management participation was binary (ever received vs. never received) and did not assess service availability, accessibility, or quality, we cannot directly attribute this gap to insufficient coverage or inequity in community health services. Instead, this finding should be interpreted as suggestive evidence of unmet support needs among chronically ill older adults in this sample.

Future perspectives: equity-oriented interventions are urgently needed. Strategies include: (1) means-tested subsidies or waivers for low-income older adults to reduce financial barriers; (2) community-based health literacy programs tailored to older adults with limited formal education; (3) proactive outreach to individuals newly diagnosed with chronic conditions to provide structured self-management support.

### 4.3 Non-significant findings: perceived threat and barriers

Contrary to the original HBM formulation (10) but consistent with more recent meta-analyses (14, 15), perceived susceptibility and perceived severity were not independently associated with health management behavior in the multivariate model. Several explanations may account for this non-significance.

First, among older adults—particularly those with existing chronic conditions—threat perceptions may already be universally high, resulting in limited variability that constrains statistical power to detect associations. Second, the HBM posits that threat perceptions are necessary but not sufficient for behavior change; they must be coupled with strong efficacy beliefs to motivate action (16, 36). Our bivariate analyses indicated that participants with chronic diseases reported significantly higher perceived susceptibility and severity, yet these perceptions did not translate into higher participation rates without corresponding self-efficacy. This pattern supports the threat-efficacy interaction hypothesis proposed by Witte's Extended Parallel Process Model (37).

Third, measurement issues cannot be discounted. Although our scale demonstrated acceptable psychometric properties, two-item subscales may not capture the full complexity of threat perceptions. Future research should employ more comprehensive measures or qualitative methods to explore how older adults conceptualize disease risk and severity in their daily lives.

Regarding perceived barriers, the finding that this variable approached but did not achieve statistical significance (*P* = 0.07) warrants deeper discussion for several reasons. First, the marginal *P*-value, combined with the expected negative direction of the effect, suggests that a true association may exist but was not fully captured due to sample size limitations. *Post-hoc* power analysis indicated that our study had 80% power to detect an odds ratio of approximately 1.45 for barriers; thus, a smaller but clinically meaningful effect (e.g., OR = 1.30) might have remained undetected. Second, the relatively low mean score for perceived barriers (2.97 ± 0.88) indicates that, on average, participants did not view health management as being associated with major obstacles. This restricted range (possible range 1–5) likely attenuated the observed correlation with behavior, consistent with statistical principles that range restriction reduces effect size estimates. Third, the operationalization of perceived barriers in this study focused on practical obstacles (e.g., cost, transportation, time), but qualitative pilot work suggested that for many older adults, barriers are often psychosocial (e.g., fear of being a burden, lack of family encouragement, embarrassment about health conditions). These psychosocial dimensions may be equally or more influential than practical barriers, yet they were not adequately captured by our scale.

The subgroup finding that older adults living alone reported significantly higher perceived barriers compared to those living with family adds another layer of complexity. This difference was not merely a matter of magnitude: qualitatively, those living alone more frequently cited “no one to remind me” and “I don't see the point if no one cares” as barriers, whereas those living with family focused on practical concerns such as transportation or clinic wait times. This suggests that for socially isolated older adults, the absence of social reinforcement transforms perceived barriers from logistical challenges into existential ones—a distinction that has important implications for intervention design. Specifically, simply reducing out-of-pocket costs or improving transportation access may be insufficient for this subgroup if the underlying issue is a lack of perceived accountability or social meaning. Our findings align with the broader literature demonstrating that social isolation is associated with adverse health outcomes and that family support serves as a critical cue to action in Chinese cultural contexts (25, 38, 39).

Future perspectives: interventions targeting socially isolated older adults should address both instrumental support (e.g., transportation assistance, appointment reminders) and, crucially, relational support (e.g., peer support groups, volunteer visiting programs that provide consistent social contact, community-based “health buddy” systems). Given our finding that psychosocial barriers predominated among those living alone, interventions should incorporate meaning-making components—such as connecting health management activities to valued social roles (e.g., “staying healthy to help neighbors” or “to remain independent for as long as possible”). Family-centered approaches that engage adult children in supporting their parents' health management may be particularly effective in collectivist cultural settings. For older adults without family nearby, community health workers or trained volunteers can serve as surrogate social anchors, providing both practical assistance and the essential social reinforcement that transforms perceived barriers from insurmountable to manageable.

### 4.4 Comparison with previous studies

Regarding regional differences, our observed participation rate (54.4%) is substantially lower than rates reported in more developed Chinese regions, such as Shanghai (approximately 72%) and Beijing (approximately 68%), and falls considerably short of rates in high-income countries, including Japan (>80%) and the United States (approximately 65%−70% for comparable age groups). These disparities likely reflect regional variations in healthcare infrastructure (e.g., density of community health stations, availability of family doctor teams, and maturity of senior care networks such as the “15-min older adult care living circle”) as well as cultural factors (e.g., stronger reliance on family support in Guangxi's traditional kinship structures vs. greater acceptance of institutionalized services in more urbanized regions). These observations underscore the importance of geographically tailored health policies rather than a one-size-fits-all approach and suggest that improving participation in less-developed regions may require context-specific strategies that address both infrastructure gaps and cultural norms.

Our findings are broadly consistent with prior HBM-based research conducted in diverse populations and settings. A meta-analysis by Carpenter (14) of 18 studies found that perceived benefits and perceived barriers were the strongest and most consistent predictors across behaviors, with self-efficacy showing particularly large effects for complex, ongoing behaviors. Our results align closely with this pattern.

Within the Chinese context, our findings corroborate and extend the work of Wang et al. (26), who reported that health literacy and self-efficacy were significant predictors of health-related quality of life among hypertensive patients in rural western China. This study makes several incremental contributions beyond prior literature. First, while most HBM-based studies in China have focused on disease-specific self-management (e.g., hypertension, diabetes), we examined general health management behaviors encompassing prevention, monitoring, and treatment adherence, providing a broader understanding of older adults' overall health engagement. Second, we rigorously validated a culturally adapted Health Belief Scale specifically for the Nanning population using confirmatory factor analysis—a methodological step often omitted in prior regional studies—thereby enhancing measurement reliability. Third, unlike previous research conducted in rural western China (e.g., Wang et al., which focused on health literacy and quality of life), our urban community sample from a provincial capital captures a distinct socioeconomic and healthcare access context, revealing that even in urban settings with relatively better resources, participation remains suboptimal and disparities persist. Fourth, we explicitly quantified the independent contributions of both psychological beliefs (benefits, self-efficacy) and structural factors (education, income) within a single model, demonstrating that health beliefs explain only part of the variance; socioeconomic resources exert effects independent of beliefs. This finding challenges purely cognitive models and suggests that interventions must address material constraints alongside belief change. However, our study differs in several important respects. First, we focused on general health management behaviors rather than disease-specific self-management, capturing a broader spectrum of preventive activities. Second, our sample was drawn from an urban community in a provincial capital, representing a different socioeconomic and healthcare access context. Third, we employed rigorous psychometric validation procedures for the Health Belief Scale, enhancing measurement reliability.

Nevertheless, our observed participation rate (54.4%) is lower than that reported in studies from more developed regions of China (40) and substantially lower than rates in high-income countries (41). This disparity underscores the observed differences in participation rates between our sample and those from more developed regions or high-income countries. However, because our study used a binary measure of health management participation (ever received vs. never received) and did not directly assess healthcare system coverage, service availability, or regional resource allocation, we cannot draw direct conclusions about systemic inequities in community health service provision. Instead, our findings highlight a descriptive gap in participation that warrants further investigation using more granular measures of service access and quality. Future research should directly compare service coverage, health literacy, and intervention reach across regions to determine whether observed participation differences reflect true systemic inequities.

## 5 Key findings summary

**Figure 4:**
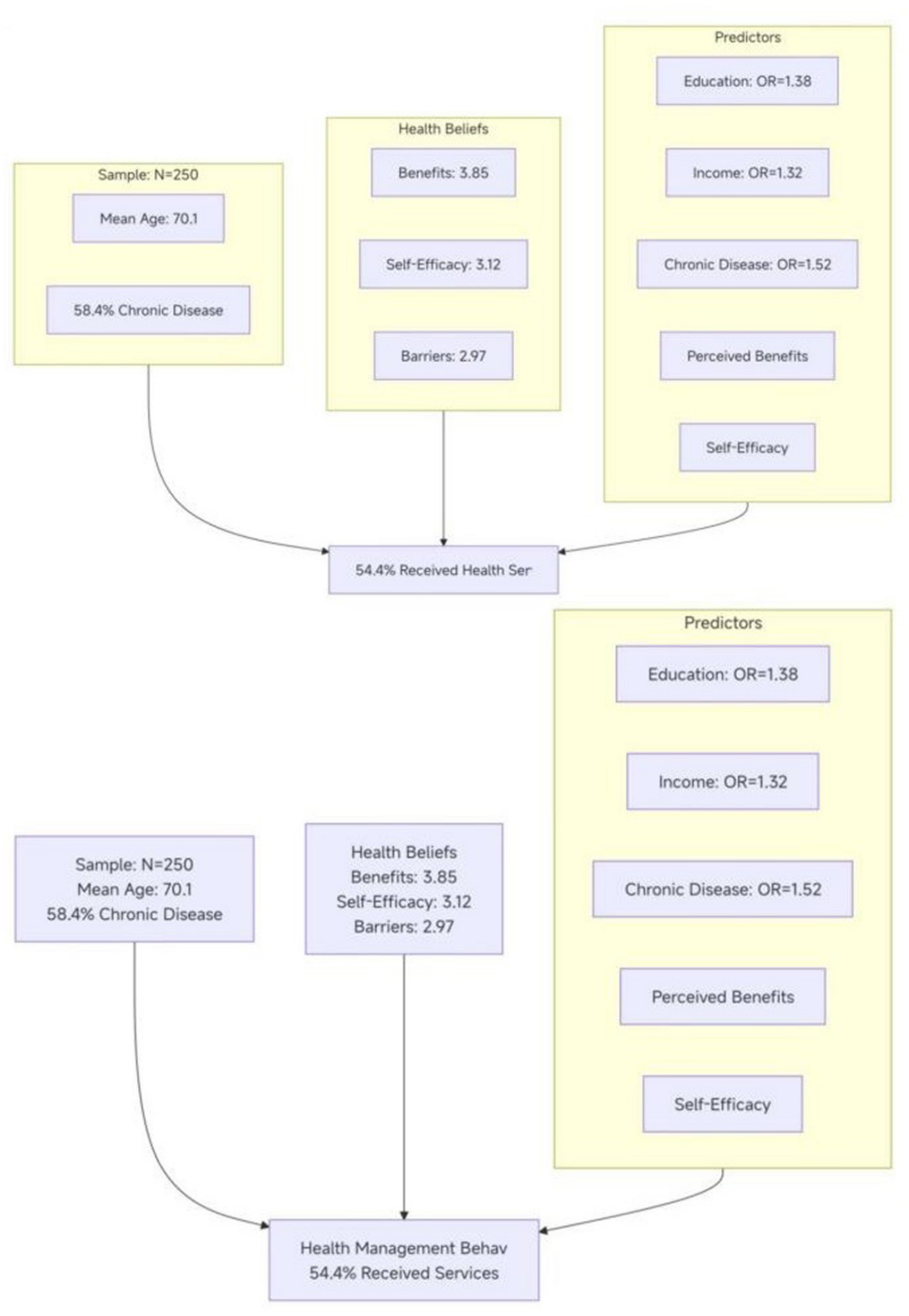
Key predictors of health management participation among community-dwelling older adults in Nanning, China.

The diagram maintains the key relationships while avoiding complex subgraphs that could cause rendering issues (Figure 4).

## 6 Strengths and limitations

### 6.1 Methodological strengths

This study has two primary strengths. First, the application of a theoretically grounded framework (HBM) enabled systematic examination of both psychological and structural determinants of health behavior. Second, the Health Belief Scale underwent rigorous cultural adaptation and psychometric validation, including confirmatory factor analysis, ensuring measurement reliability and validity.

### 6.2 Limitations

Several limitations must be acknowledged when interpreting these findings.

First, the sample size (*N* = 250) is relatively small, which limits the detection of small effects and subgroup interactions, as reflected in the wide confidence intervals for some estimates (e.g., education: OR = 1.38, 95% CI: 1.01–1.90).

Second, the use of convenience sampling from a single urban community (Nanning) substantially limits generalizability. Our findings may not apply to rural older adults, institutionalized populations, or older adults in other Chinese cities.

Third, the cross-sectional design precludes causal inferences, and longitudinal studies are needed to establish temporal precedence.

Fourth, measurement limitations include the use of only two items per health belief dimension and a dichotomous measure of health management behavior (ever received vs. never received), which does not capture frequency, consistency, or quality of participation.

Fifth, self-report bias may have introduced recall and social desirability biases, and unmeasured confounders (e.g., social support networks, health literacy, depressive symptoms) warrant future investigation.

## 7 Conclusions and implications

### 7.1 Conclusions

This study provides empirical evidence that health management behaviors among community-dwelling older adults in Nanning, China, are significantly influenced by perceived benefits, self-efficacy, educational attainment, income level, and chronic disease status. The overall participation rate observed in this study was low (54.4%), and the significant socioeconomic disparities indicate that the current community health services have not yet achieved universal participation in the recommended health management practices, especially among subgroups with lower socioeconomic status (those with lower education levels and income).

The findings support the utility of the HBM as a framework for understanding health behaviors in this population, while also highlighting the need to integrate structural determinants into behavior change theories. Perceived threat dimensions (susceptibility and severity) were not independent predictors, suggesting that interventions focused solely on fear arousal are unlikely to be effective without concurrent efforts to enhance efficacy beliefs and reduce practical barriers.

### 7.2 Implications for policy and practice

Based on our findings, we propose a multi-level intervention framework targeting individual beliefs, interpersonal processes, and structural barriers:

1. Individual-level interventions:
  - Develop and implement community-based health education programs that explicitly communicate the benefits of health management using simple, vivid, and culturally resonant messaging.
  - Integrate self-efficacy enhancement techniques into routine community health services, including mastery experiences (e.g., guided practice of blood pressure self-monitoring), vicarious experiences (e.g., peer testimonials), verbal persuasion (e.g., encouraging messages from healthcare providers), and physiological feedback (e.g., demonstrating improved health indicators).
  - Provide personalized risk communication for older adults with chronic conditions, translating epidemiological risk statistics into personally meaningful information.
2. Interpersonal-level interventions:
  - Engage family members, particularly adult children, as partners in health management through family health education sessions and caregiver support programs.
  - Establish peer support networks and community health volunteer programs to reduce social isolation and provide role models for health management behaviors.
  - Train community health workers to deliver culturally tailored health coaching using motivational interviewing techniques.
3. Structural-level interventions:
  - Expand financial protection mechanisms, including medical insurance reimbursement for preventive services and subsidies for low-income older adults.
  - Improve service accessibility through extended clinic hours, home-based service delivery, transportation assistance, and integration of health management services with other community services (e.g., senior centers, meal programs).
  - Invest in health literacy-friendly environments, including clear signage, plain language health materials, and navigation assistance within community health centers.
  - Strengthen the primary care system by enhancing the capacity of community health service centers to deliver comprehensive, coordinated, and patient-centered chronic care.

### 7.3 Future perspectives for research

To advance knowledge in this field, future research should prioritize the following directions should:

1. Employ longitudinal designs to establish causal relationships and examine how health beliefs and behaviors evolve over time, particularly during health transitions (e.g., new chronic disease diagnosis, loss of spouse, relocation).
2. Utilize mixed methods approaches combining quantitative surveys with in-depth qualitative interviews to understand the lived experiences, decision-making processes, and contextual factors shaping health management among diverse subgroups of older adults.
3. Develop and test theory-based interventions using rigorous experimental designs (e.g., randomized controlled trials) to evaluate the effectiveness of self-efficacy enhancement programs, health literacy interventions, and service delivery innovations.
4. Conduct comparative research across different regions of China and across countries to identify context-specific and universal determinants of health management behaviors among aging populations.
5. Investigate digital health interventions, including mobile health applications and telehealth services, as potential strategies to reach older adults with limited mobility or those residing in underserved areas.
6. Examine the role of community social capital—trust, reciprocity, and civic engagement—in facilitating collective health management behaviors among older adult populations.

## Data Availability

The original contributions presented in the study are included in the article/Supplementary material, further inquiries can be directed to the corresponding author.
