# Supplementary material for: Predictors of health management participation in community-dwelling older adults: a cross-sectional study based on the health belief model
Source: Front Public Health. 2026 Jun 11;14:1797890. doi: 10.3389/fpubh.2026.1797890 (PMC13295613; doi:10.3389/fpubh.2026.1797890)
Supplement: Supplementary file 1 [file Table_1.doc]

# **Appendix:**

**Survey on Elderly Health Management Awareness**
**Dear Grandparents,
Hello! This questionnaire aims to study the factors that influence the health management needs of the elderly. There are no right or wrong answers; please respond based on your actual situation and feelings. The survey results will be used for research purposes only, and your personal information will never be disclosed. You can fill it out with confidence! Thank you sincerely for your cooperation!**

1. Basic information (please check "√" or fill in the relevant content in "_" before the reasonable option)
1. Your gender [multiple choice] *
○ Male ○ Female
2. Your age: [Multiple choice] *
○ 60-65 years old ○ 66-70 years old ○ 71-75 years old ○ 76 years old and above
3. Your education: [Multiple choice] *
○ Elementary school and below ○ Junior high school ○ High school/technical secondary school ○ College and above
4. Your monthly income (yuan): [Multiple choice] *
○＜1000 ○1001-2000 ○2001-3000 ○>3000
5. Do you have a chronic disease (e.g., diabetes, hypertension, etc.) [Multiple choice] *
○Yes ○ No
6. Your residence: [Multiple choice] *
○ Living alone ○ Living with family ○ Nursing home
2. Health management cognition (4 questions in total, please type "√" in the corresponding option)
7. Do you have access to health management knowledge? [Multiple Choice] *
○Yes ○No (Skip to question 9) (Please skip to question 9)
8. The main way for you to acquire health management knowledge (multiple choice): [Multiple choice question] *
□ Community advocacy □ Health care workers □ TV/radio □ Recommendations from relatives and friends □ Online platforms □ Books/newspapers
□ Other: _________________*
9. Do you think health management is important for preventing chronic diseases? [Multiple Choice] *
○ Very important ○ Important ○ General ○ Not important ○ Very unimportant
10. Do you know the risk factors for chronic diseases (e.g., high-salt diet, lack of exercise)? [Multiple Choice] *
○ All I understand ○ I understand part ○ I don't understand
3. Health management behavior (4 questions in total, please type "√" in the corresponding option)
11. Have you received health management services (such as physical examinations, health lectures, etc.) in the community or medical institutions? [Multiple Choice] *
○Yes ○ No
12. Do you have regular health check-ups? [Multiple Choice] *
○ Once every six months ○ Once a year ○ Occasionally ○ Never
13. Do you follow your doctor's health advice (e.g., dietary control, regular medication)? [Multiple Choice] *
○ Always ○ Often ○ Occasionally ○ Rarely ○ Never
14. If you do not participate in health management, the main reasons are: [Multiple choice questions] *
□ financial burden □ limited mobility □ lack of knowledge □ mistrust effect □ unaccompanied □ other
4. Health Belief Scale (10 questions in total, please choose according to feelings, 1 = strongly disagree, 5 = strongly agree) dimension question 12345
1. I think I may suffer from chronic diseases due to bad habits in the future [Multiple Choice] *
○1 ○2 ○3 ○4 ○5
2. I am concerned that chronic illness will affect my ability to live daily life. [Multiple Choice] *
○1 ○2 ○3 ○4 ○5
3. Worsening chronic illness can prevent me from living independently. [Multiple Choice] *
○1 ○2 ○3 ○4 ○5
4. Chronic diseases may increase the financial burden on the family. [Multiple Choice] *
○1 ○2 ○3 ○4 ○5
5. Health management can reduce my risk of illness. [Multiple Choice] *
○1 ○2 ○3 ○4 ○5
6. Regular check-ups give me more confidence in my health. [Multiple Choice] *
○1 ○2 ○3 ○4 ○5
7. Health management takes too much time and money. [Multiple Choice] *
○1 ○2 ○3 ○4 ○5
8. I don't know how to get effective health management information. [Multiple Choice] *
○1 ○2 ○3 ○4 ○5
9. I am confident that I will develop and adhere to a health management plan [Multiple choice] *
○1 ○2 ○3 ○4 ○5
10. Even if I have limited mobility, I can still help manage my health through my family. [Multiple Choice] *
○1 ○2 ○3 ○4 ○5
